# Diastolic dysfunction in spontaneous type 2 diabetes rhesus monkeys: a study using echocardiography and magnetic resonance imaging

**DOI:** 10.1186/s12872-015-0046-9

**Published:** 2015-06-26

**Authors:** Can Qian, Li Gong, Zunyuan Yang, Wei Chen, Yushu Chen, Ziqian Xu, Bing Wu, Chungui Tang, Fabao Gao, Wen Zeng

**Affiliations:** 1 Sichuan Industrial Institute of Antibiotics, Chengdu, China; 2 Sichuan PriMed Shines Bio-Tech Co., Ltd, Chengdu, China; 3 Department of Radiology, West China Hospital, Sichuan University, Chengdu, China; 4 Department of Radiology, Yaan People’s Hospital, Yaan, China

## Abstract

**Background:**

Diastolic heart failure is a common and deadly complication of diabetes mellitus, with the development of diabetic cardiomyopathy as one of the key determinants of the disease’s complex pathology. The cause of the association is unknown and has no approved therapy strategies as of yet. However significant advances in this area may come from studies on suitable animal models.

**Methods:**

A total of 25 male rhesus monkeys (12-16 years, 9-13 kg) were enrolled. Fifteen of them were diagnosed as spontaneous type 2 diabetes mellitus (T2DM, FPG ≥ 104 mg/dl, HbA1c: 4.7-5.5 %, diabetes duration: 1-4 years). The other 10 monkeys were non-diabetic (ND, FPG < 90 mg/dl). Echocardiography and cardiac magnetic resonance were used for evaluating the cardiac structure and function. One T2DM monkey with impaired diastolic function and another ND monkey were both sacrificed to gain the necessary pathology and protein expression studies displayed here.

**Results:**

Six out of 15 T2DM rhesus monkeys were diagnosed with diastolic dysfunction (DD) by echocardiography. Additionally, no abnormalities were found in the group which we determined as the ND monkeys. The six DD monkeys all showed low e’ velocity and decreased e’/a’ ratio, among which three of them showing decreased E/A ratio and the other 3 having elevated E/A ratio, this appears to be similar to the impaired relaxation pattern and pseudonormal pattern found in human patients respectively. The EF and FS of monkeys with pseudonormal pattern decreased significantly compared with ND subjects. A CMR study showed that LVID at end systole of 5 DD monkeys is significantly longer than that of 3 ND monkeys. Of great interest, myocardium lesions and mitochondria impairments and increased expression of AGEs and caspase-3 were found in a sacrificed DD subject.

**Conclusion:**

The changes in the imaging and physiological markers of spontaneous T2DM rhesus monkeys are similar to those key markers found in human type 2 diabetes and diastolic dysfunction. This monkey model could help the medical community and us to understand the pathology of this debilitating disease and serve as a beginning to explore important measures to prevent and treat diabetic cardiomyopathy.

## Background

Cardiovascular diseases (CVD) are the leading causes of death and disability among people with diabetes. At least 65 % of people with diabetes die from certain forms of cardiovascular diseases [1]. As a major outcome of diabetes, cardiac failure is the most severe comorbidity [2]. A high prevalence of cardiac failure is seen in individuals with diabetic cardiovascular complications, therefore diabetic cardiomyopathy (DCM) is one of the key determinants in understanding and controlling the dire effects of the onset and progression of diabetes mellitus [3].

DCM has been defined as ventricular dysfunction that occurs in diabetic patients independent of a recognized cause, such as coronary artery disease (CAD) or hypertension [4]. Additionally, DCM is marked by diastolic dysfunction (DD) early on the disease’s progression [5–7] that leads to the loss of contractile (systolic) function as time passes by [7–9]. The pathogenesis of this ventricular dysfunction remains unknown, but several hypotheses have been proposed, including autonomic dysfunction, metabolic derangements, abnormalities in ion homeostasis, alteration in structural proteins and the presence of interstitial fibrosis.

Alterations of certain biomarkers have also been reported to play key roles in the development of cardiac dysfunction and are therefore important to monitor. For example, advanced glycosylation end products (AGEs) can covalently crosslink and biochemically modify collagen structures in diabetes induced myocardial stiffness [10]. Another biomarker of interest is caspase-3 activation triggered by ROS generation in hyperglycemia-induced cardiac cell apoptosis in vivo [11]. Monitoring PKA-mediated phosphorylation of cMyBP-C is also helpful as it is linked to the modulation of cardiac contractions [12]. Determinations of these biomarkers contribute to providing the evidence of impairments by monitoring the abnormal effects in different metabolic pathways.

Significant advances in diabetic-related diseases may come from the studies on suitable animal models. Abnormalities in cardiac functionality and structure in diabetic subjects have been demonstrated in a variety of type 1 and type 2 diabetic rodent models [13–18]. In most studies of type 1 diabetes mellitus, diabetes is induced after administration of the pancreatic beta-cell toxin streptozotocin (STZ). Additionally, most studies of type 2 diabetes mellitus have been performed in genetic models demonstrating obesity and insulin resistance such as the Zucker fatty rat or db/db mice, both of which have mutations that impair leptin receptor signaling, or ob/ob mice, which lack leptin [4]. STZ induced and transgenic/gene knock-out diabetic rodent models may have advantages in studying some particular mechanisms. However, as for the complicated pathogenesis of DM, neither the rats nor the mice appear to have similar cardiac dysfunction that stimulates diabetic cardiomyopathy in diabetic humans.

Compared with other animal models, rhesus monkeys (*Macaca mulatta*) are more similar to humans in physiology and to the susceptibility of known metabolic diseases in human patients [19]. Though naturally occurring spontaneous type 2 diabetes has been extensively studied in rhesus [20–22], diabetic cardiovascular complications in rhesus monkeys have not been adequately discerned. In our previous work, a certain number of spontaneous T2DM rhesus monkeys have been screened and found within our colony, and their DM related parameters were demonstrated to be similar to humans [23]. Therefore, to investigate cardiac characteristics of those subjects, T2DM and age matched non-diabetic (ND) rhesus monkeys were enrolled in this study. Multiple complementary image techniques such as M-mode echocardiography, pulsed wave Doppler ultrasound, tissue Doppler imaging and cardiac magnetic resonance (CMR) were used to assess and categorize the DD group. Furthermore, light-scope was utilized to observe the microstructure within major organs to help us visualize the pathology present. Transmission electron microscope (TEM) was also used to observe the myocardium mitochondrial ultrastructure. These images were obtained through the application of immunohistochemical staining to determine and show the cardiac proteins of interest.

## Methods

### Animals

Fifteen previously screened spontaneous T2DM rhesus monkeys from ages 12 to 16 years, with 1–4 years’ duration of diabetes [23] were matched with 10 ND rhesus monkeys of a similar age for testing. The criteria used for the rhesus monkey selections were guided by our previous work [23] along with similar criteria for rhesus monkeys [24, 25]. Monkeys were singly housed in a climate controlled room at 19 °C to 26 °C (66 °F to 79 °F), with a relative humidity of 50 % ± 20 %. The rate of ventilation was 10 times/h and the lighting cycle was 12 (day)/12 (night) hours. Monkeys were continually fed with mild high-calorie diet (containing 18 % protein, 60 % carbohydrates, 14 % water, and 8 % fat) approximately 300 g/day; a daily allotment of apples or vegetable was also provided. Tap water was provided as drinking water ad libitum. The monkeys were maintained in conformity with the requirements of “the National Institutes of Health Guide for the Care and Use of Laboratory Animal” of the United States, and all experimental protocols had been reviewed and approved by the Institutional Animal Care and Use Committee of Sichuan PriMed Group Co., Ltd.

### Trial arrangements

There was a 4-week long acclimation period before the initial image study, during which gravimetry and metabolic profiles of all enrolled monkeys were acquired, see Table 1. Fasting plasma glucose (FPG) were determined semi-monthly for four weeks. Hemoglobin A1c (HbA1c), 2 h post-challenge plasma glucose (2hPPG), total cholesterol (TC), triglyceride (TG), low density lipoprotein cholesterol (LDL-c) and high density lipoprotein cholesterol (HDL-c) were determined at the end of the acclimation period. Body weight (BW), crown-rump length (CRL) of all monkeys were measured after the last blood collection to calculate BMI. Blood pressure of all the monkeys were measured before each image examination. Echocardiography were performed on all monkeys in 48 h after the last blood collection. Cardiac magnetic resonance (CMR) were performed sequentially on some of the monkeys. One monkey with moderate diastolic dysfunction, together with one ND monkey was sacrificed for histopathological and protein study. Some of the pathology was detailed by use of transmission electron microscope for observing microstructure and ultrastructure. Immunohistochemistry staining were performed for the protein studies.

**Table 1:** Gravimetry and metabolic profile of monkeys enrolled for the study showing measurements in the acclimation period

Groups		ND	T2DM
		(*n* = 10)	(*n* = 15)
Age (year)		12-16	12-16
BW (kg)		9.2 ± 1.9	11.3 ± 1.2
BMI		28.8 ± 2.8	38.0 ± 2.8*
Diabetes Duration (year)		0	1-4
FPG (mg/dL)	day 14	82.6 ± 10.3	119.8 ± 12.9*
	day 28	78.6 ± 9.8	113.9 ± 8.6*
2hPPG (mg/dL)		98.1 ± 16.4	137.8 ± 24.5*
HbA1c (%)		4.2 ± 0.4	5.1 ± 0.2*
TC (mg/dL)		131.6 ± 27.1	140.0 ± 17.6
TG (mg/dL)		44.4 ± 22.9	51.0 ± 30.9
LDL-c (mg/dL)		46.4 ± 11.6	53.7 ± 13.5
HDL-c (mg/dL)		61.4 ± 10.8	62.1 ± 11.6
SBP (mmHg)		129 ± 6	154 ± 7*
DBP (mmHg)		114 ± 7	109 ± 19

*p < 0.05, compared with ND group

### Laboratory measurements

FPG, 2hPPG, HbA1c and lipid parameters were measured as we previously performed [23, 26]. Body mass index (BMI) was calculated using body weight divided by the square of CRL. Blood pressures were measured by an intelligent non-invasive blood pressure monitor while the monkeys were seated upon a monkey chair.

### Image examination

Echocardiography: All monkeys were fasted overnight prior to echocardiography examination. Monkeys were sedated by ketamine hydrochloride (Bioniche Teoranta) at a dosage of 10 mg/kg i.m. and the coat of monkey was bilaterally shaved from the cervical part to the navel, and rinsed copiously with water in order that acoustic coupling was obtained using ultrasound gel. Transthoracic echocardiography was performed by using a commercially available ultrasound system (Mindray M7vet) with the same investigator taking the measurements with the monkeys placed in a dorsal decubitus position while in a stable sedation state. All measurements were performed around the midday to avoid the influence of circadian rhythm on the left ventricular diastolic function [27]. All echographic acquisitions were obtained according to the recommendations of the American Society of Echocardiography and digitally stored from at least three consecutive heartbeats for offline analysis.

Pulsed-wave Doppler measurements: Blood flow velocity was recorded at the mitral diastolic inflow at the level of the mitral leaflet tips from the apical 4-chamber view. The following velocities were observed and recorded according to the current standards of the practice of echocardiography: E wave, A wave and E/A ratio. Early filling deceleration time (EDT) and isovolumic relaxation time (IVRT) were measured and averaged from 3 consecutive beats. Pulmonary venous flow (PVF) was recorded from the same acoustic window, placing a sampling volume 1 cm into the right upper pulmonary vein. Peak systolic (S), diastolic (D), and atrial reversal (AR) velocities were measured and averaged from 3 consecutive beats.

Conventional M-mode Doppler ultrasound: Real-time two-dimensional targeted M-mode echocardiograms of the LV minor axis was taken at the papillary muscle level to measure the EF and FS. EF = (EDV-ESV)/EDV. In this study, the values of EDV and ESV were measured by using the Teichholz-corrected formula in standard software included in the ultrasound equipment. EF and FS were calculated automatically by the system when relative cardiac borders were set.

Tissue Doppler imaging: to perform this, the ultrasound equipment was switched into tissue Doppler imaging mode, sample volume was located at the septal side of the mitral annulus from the apical 4-chamber view. Early (e’) and late (a’) diastolic mitral annulus velocities and the ratio of early to late peak velocities (e’/a’) were obtained.

### Cardiovascular magnetic resonance

In one week after echocardiography examination, CMR imaging was performed with a 3.0-T whole-body MR system (MAGNETOM Trio, Siemens Medical Solutions, Erlangen, Germany) on four ND monkeys and only five DD monkeys (anesthesia failure happened to one monkey with moderate DD). Intravenous propofol were used for anesthesia induction, and inhalation isoflurane were used for anesthesia maintenance. Data were acquired during end-inspiratory breath holding. After scout images, TrueFISP cine sequence (TE/TR = 11/500 ms; field of view (FOV) = 20 cm x 20 cm, NEX = 2; matrix = 256 x 256; flip angle, 50°; slice thickness = 2 mm; slice gap = 2 mm) with retrospective ECG-gating was used to acquire dynamic cine loops of the LV for function analysis. The LV was imaged in its entirety from the base to the apex in 9–12 short-axis cine images without inter-slice gaps or overlaps.

Cine function analysis was performed off-line with commercial software (Argus, Siemens Medical Solutions). At the same mid-ventricular level, endocardial and epicardial boundaries were traced semi-automatically during the end-diastolic and end-systolic phases in order to obtain the dimensional parameters: LVIDd, LVIDs, SWTd, SWTs, AWTd, AWTs, PWTd, and PWTs. Left ventricular cine images were analyzed by an experienced radiologist who was blinded to the echocardiographic results.

### Light scope and transmission electron microscopy (TEM) observation

Extracted heart, liver, kidney and pancreas of a DD monkey were processed for H & E staining. The ultrastructure of myocardial mitochondria were observed by transmission electron microscope (TEM) as we previously did [28]. Fresh tissues (cardiac muscle) were cut into 1 mm cubes, which were fixed in 3 % glutaraldehyde for 2 h, and then fixed in 1 % osmium tetroxide, with stepwise dehydration in graded acetone, then after being infiltrated and embedded, tissue was polymerized in EPON 812. The semi-thin sections were optically positioned and further sectioned with ultramicrotome into 50–60 nm pieces, which were collected on copper grids, double-stained with uranyl acetate and lead citrate, and then observed under Hitachi H-600IV transmission electron microscope and photographed.

### Immunohistochemistry assessment for protein study

Five-micron serial paraffin sections of LV were used to stain for AGEs, caspase-3 and phosphorylated cMyBP-C. Sections were dewaxed in xylene, rehydrated through a graded series of ethanol, washed in distilled water and phosphate-buffered saline (PBS), and then blocked for endogenous peroxidase by incubation with 3 % H_2_O_2_ in methanol for 15 min. The sections were subjected to antigen retrieval procedure by microwaving in 0.01 M pH 6.0 sodium citrate buffer. Additional washing in PBS was performed before the next 30 min of incubation at 37 °C in 10 % normal goat serum. The sections were incubated overnight at 4 °C with diluted (1:100) primary antibodies. The antibodies used were polyclonal goat anti-rabbit AGEs antibodies, goat anti-rabbit caspase-3 antibody and goat anti-rabbit phosphorylated cMyBP-C antibody (Boster, China). For negative controls, the sections received PBS in place of the primary antibody. After washing in PBS, the sections were exposed to a 1 % biotinylated secondary antibody goat and anti-rabbit IgG (Boster, China) for 1 h at 37 °C. The sections were then incubated with the HRP-streptavidin (Boster, China) for 30 min at 37 °C. To visualize the immunoreaction, the sections were immersed in blue diaminobenzidine hydrochloride (DAB). The reaction was monitored microscopically and stopped by the immersion into distilled water as soon as a brown color staining was clearly visualized.

### Statistical analysis

Statistical analysis was performed using SPSS 19.0 software. The data is presented as the mean ± SD unless otherwise specified. Comparison among the two groups of subjects for various parameters was carried out by one-way analysis of variance. When normality and/or equal variance testing conditions were not met, the Kruskal-Wallis rank test and/or the Dunn's test for multiple comparisons were used, respectively. A p value less than 0.05 was considered statistically significant.

## Results

### Gravimetry and metabolic profile

Hyperglycemia and elevated HbA1c percentage were confirmed in all spontaneous T2DM monkeys enrolled (Table 1). In terms of both of the FPG determinations, T2DM group showed significantly higher FPG level compared with the ND group. All T2DM monkeys enrolled not only met our criteria for T2DM, but also showed relatively stable FPG in the acclimation period. 2hPPG and HbA1c percentage in the T2DM group were also significantly higher than those in ND group (137.8 ± 24.5 *vs* 98.1 ± 16.4 mg/dL for 2hPPG, p < 0.05; 5.1 ± 0.2 *vs* 4.2 ± 0.4, %, for HbA1c, p < 0.05). Obesity was observed in all T2DM monkeys, whose BMI average body weight was larger than that of ND group (38.0 ± 2.8 *vs* 28.8 ± 2.8, kg/m^2^, p < 0.05), while there was no significance in body weight (11.3 ± 1.2 *vs* 9.2 ± 1.9, kg). There were no significant differences between groups in their lipid profiles. Average systolic blood pressure of the diabetic monkeys was found to be higher than that of the ND monkeys (154 ± 7.0 *vs* 129 ± 5.6, mmHg, p < 0.05).

### Echocardiography findings

**Fig. 1:**
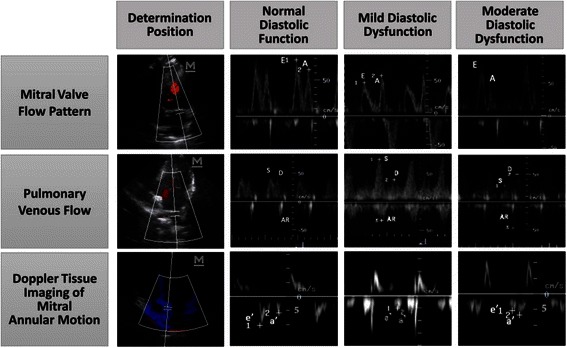
Representative images of echo patterns of mitral inflow, pulmonary venous flow and mitral annulus velocity from normal to moderate diastolic dysfunction in rhesus monkeys

The monkeys tolerated the anesthesia and procedure without difficulty. There were no post study deaths. Echocardiograms with high definition were acquired from all monkeys, representative echo images were shown in Fig. 1. Six out of 15 T2DM monkeys were found having manifestations that are similar to those of human patients with diastolic dysfunction. These six monkeys all showed low e’ velocity and a decreased e’/a’ ratio. Among these six monkeys, three showed decreased E/A ratio (below 0.9), which was similar to the echographic characteristics of mild diastolic dysfunction in human patients. While another three monkeys showed increased E/A ratio (above 1.3) accompanying abnormalities of the TDI parameters described above, which were similar to the manifestations of moderate diastolic dysfunction in human patients. Since there are no criteria for diastolic function grading in monkeys, we attempt here to classify all monkeys enrolled into 4 groups: ND, T2DM with preserved DD, mild DD (considered as impaired relaxation) and moderate DD (pseudonormal pattern), for the following analyzes.

**Fig. 2:**
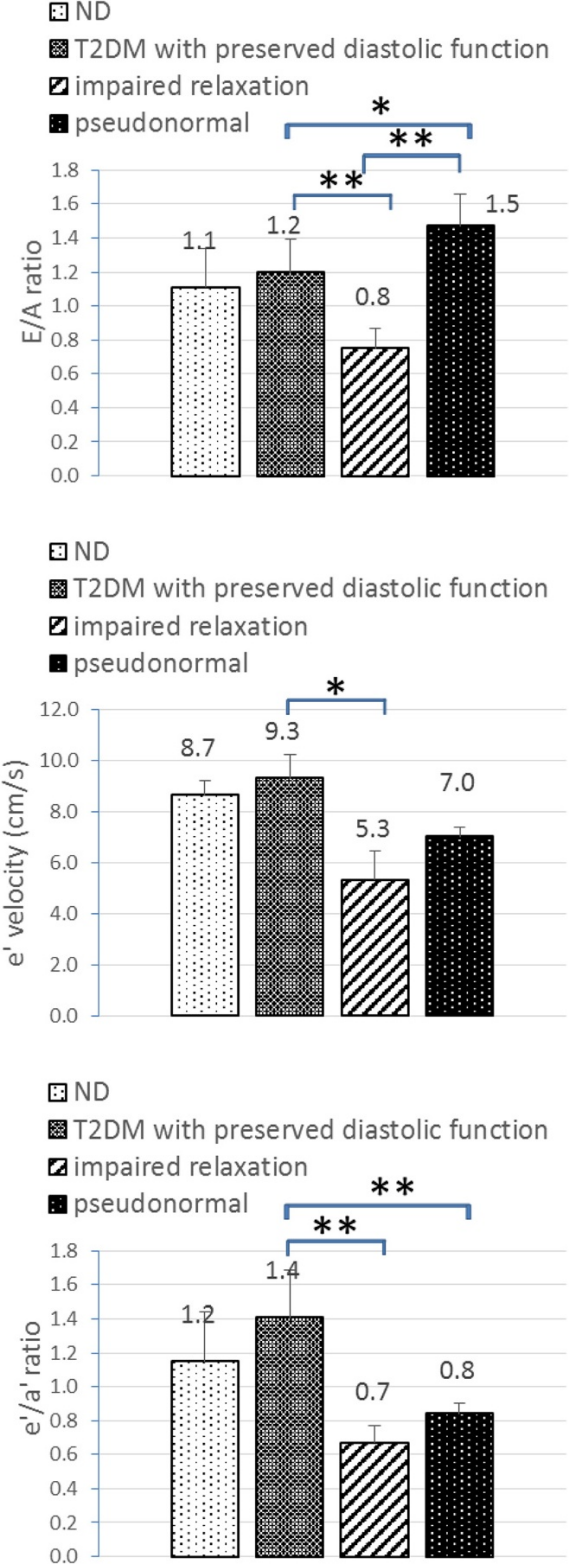
Diastolic function related parameters measured by echocardiography. *p < 0.05, **p < 0.01

In ND (n = 10) and T2DM with preserved diastolic function (n = 9) subjects averaged E/A ratio for both groups were slightly greater than 1 (1.1 ± 0.2 and 1.2 ± 0.2, see Table 2, Fig. 2), while E/A ratio significantly decreased in mild DD group (0.8 ± 0.1, n = 3) but increased in moderate DD group (1.5 ± 0.2, n = 3). Mitral annulus velocity e’ were 8.7 ± 0.6 cm/s for the ND group and 9.3 ± 0.9 cm/s for the T2DM with preserved diastolic function group, while e’ velocity decreased in DD subjects: 5.7 ± 1.7 cm/s for the mild DD group and 7.7 ± 1.6 cm/s for the moderate DD group. Significance was only found between the T2DM with the preserved diastolic function group and the mild DD group (p < 0.05). e’/a’ ratio was found to be 1.2 ± 0.3 for the ND group and 1.4 ± 0.3 for the T2DM with preserved diastolic function group, however this parameter decreased below 1 in the DD subjects: 0.7 ± 0.1 for the mild DD group (p < 0.01 compared with the T2DM group with preserved diastolic function) and 0.8 ± 0.1 for the moderate DD group (p < 0.01 compared with the T2DM with preserved diastolic function group).

**Table 2:** Echocardiographic measurements in 10 ND monkeys and 15 spontaneous T2DM monkeys

parameters	normal	T2DM with preserved diastolic function	impaired relaxation	pseudonormal pattern
	(*n* = 10)			
			(*n* = 3)	
				(*n* = 3)
		(*n* = 9)		
transmitral flow				
E/A	1.1 ± 0.2	1.2 ± 0.2	0.8 ± 0.1*	1.5 ± 0.2*#
EDT (ms)	70 ± 12	66 ± 18	92 ± 20*	75 ± 10
IVRT (ms)	48 ± 7	51 ± 10	55 ± 16	49 ± 19
pulmonary venous flow				
S(cm/s)	46.8 ± 6.1	48.5 ± 11.0	61.4 ± 9.0*	38.2 ± 2.7#
D(cm/s)	43.8 ± 11.0	45.9 ± 10.8	37.1 ± 2.0	56.9 ± 4.5*#
AR(cm/s)	43.7 ± 11.2	42.1 ± 7.9	54.7 ± 13.1	44.4 ± 2.0
myocardial velocities				
e’(cm/s)	8.7 ± 0.6	9.3 ± 0.9	5.7 ± 1.7*	7.0 ± 0.4
e’/a’	1.2 ± 0.3	1.4 ± 0.3	0.7 ± 0.1*	0.8 ± 0.1*
LA diameters				
LAAD (mm)	16.8 ± 1.0	15.1 ± 1.1	15.1 ± 2.1	15.7 ± 1.8
LATD (mm)	18.4 ± 0.7	17.8 ± 3.4	16.2 ± 2.0	16.8 ± 3.8
LAVD (mm)	21.6 ± 2.1	21.0 ± 2.2	19.3 ± 3.0	22.0 ± 5.9

*p < 0.05 compared with the T2DM with preserved diastolic function group

#*p* < 0.05 compared with the impaired relaxation group

The mild DD group had the longest average EDT (See Table 2). Significant difference was found between the mild DD group and the T2DM with preserved LV function group (92 ± 20 *vs* 66 ± 18, ms, p < 0.05). However, the difference between mild DD group and the moderate DD group was not significant. As for the IVRT, no significances were found among any of the groups.

**Fig. 3:**
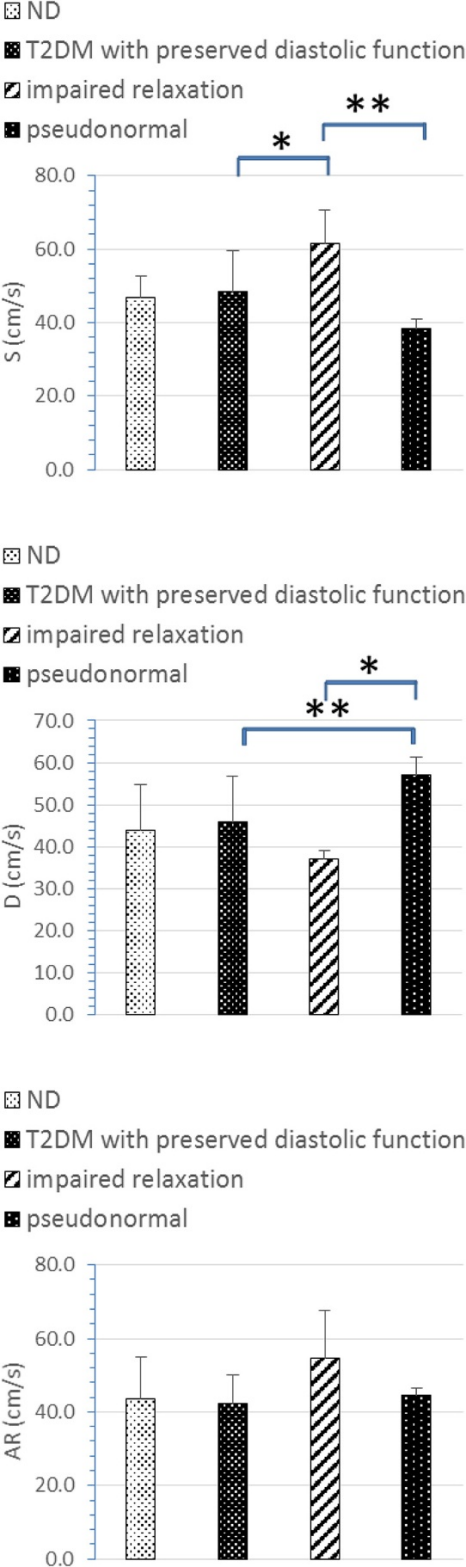
Pulmonary venous flow parameters measured by echocardiography. *p < 0.05, **p < 0.01

As for pulmonary venous flow pattern, monkeys in the ND group and T2DM with preserved LV function group showed similar levels of S, D and AR (See Table 2 and Fig. 3). In normal PVF patterns, values of S/D were around 1. In monkeys with mild DD, we have the highest peak S (61.4 ± 9.0 cm/s) and lowest D (37.1 ± 2.0 cm/s), resulting in the highest S/D ratio in this group. However, peak S decreased but D increased in the group with pseudonormal pattern, which lead to S/D < 1. As for PV AR velocity, though the highest AR waves were seen in the mild DD group, nonetheless, the differences among groups were not statistically significant.

**Fig. 4:**
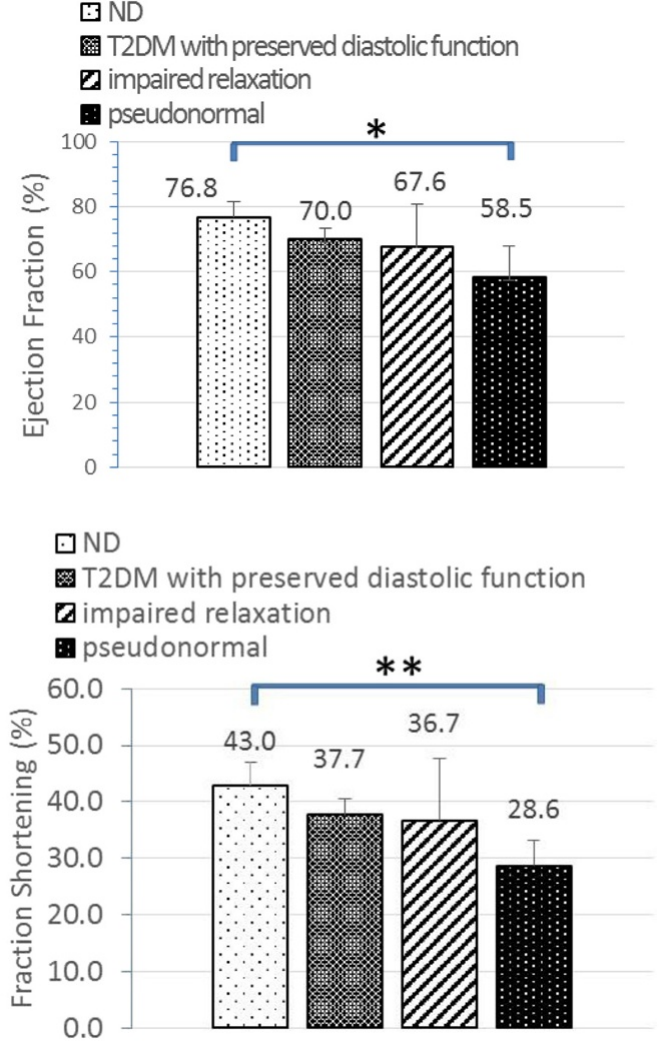
Systolic function related parameters measured by echocardiography. *p < 0.05

M-mode Doppler ultrasound was used for the measurement of left ventricular contractile capacity. Left ventricular systolic function related parameters EF and FS share similar characteristics (Fig. 4). They showed a tendency to decrease in accordance with the following order: ND, T2DM with preserved diastolic function, mild DD and moderate DD. Though there were significant differences between the moderate DD group and ND group (EF: 63.1 ± 7.0 *vs* 76.8 ± 4.9, %, p < 0.05; FS: 28.6 ± 4.5 *vs* 43.0 ± 4.0, %, p < 0.001) for these two parameters, whether they were still within normal physical limits still needs to be further studied.

### Cardiac magnetic resonance findings

**Fig. 5:**
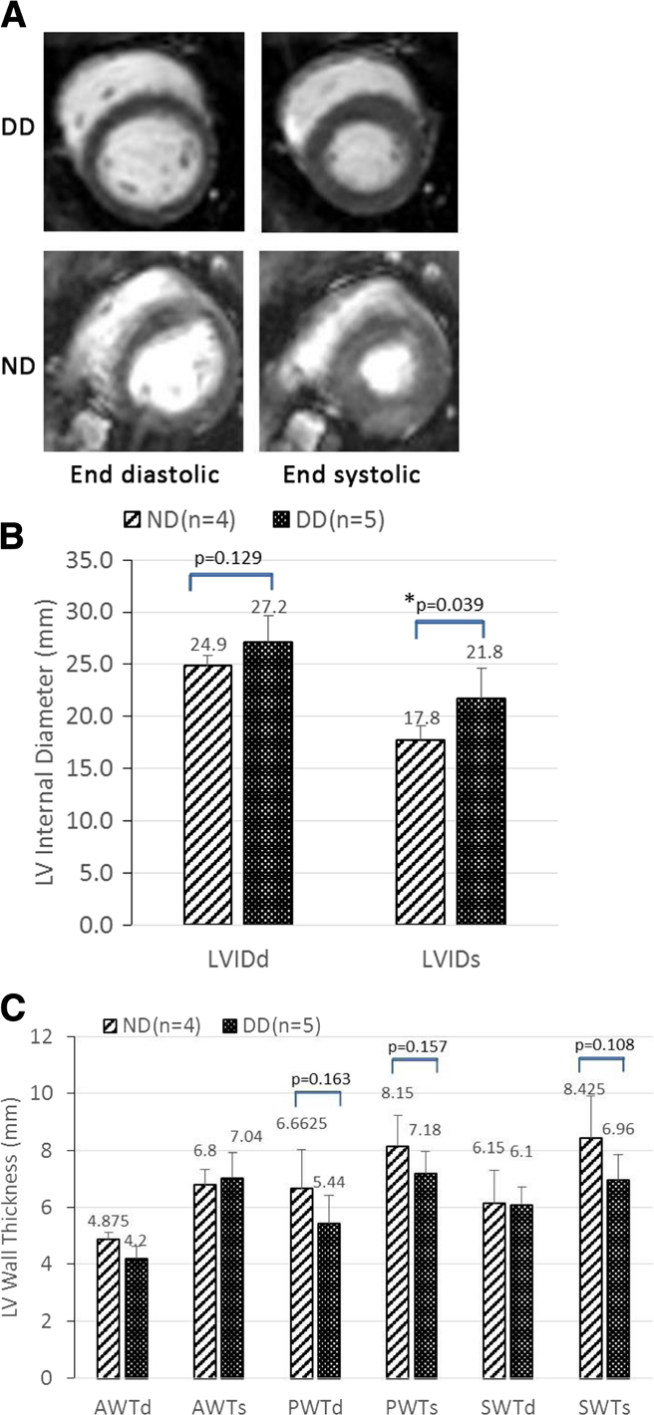
Cardiac cine-MR results measured from 5 DD monkeys and 4 ND monkeys. **a** End-diastolic (left) and end-systolic (right) cine-MR images in a mid-ventricular slice from a DD (top) and an ND (bottom) monkey. **b** Left ventricular internal dimension measured at both end diastole and end systole. **c** Left ventricular wall thickness measured at both end diastole and end systole

Left ventricular cine images at the mid-ventricular level were obtained from 5 DD monkeys and 4 ND monkeys who have been through the echocardiographic examination (Fig. 5). LV internal diameter (LVID), anterior, posterior and septum wall thickness (AWT, PWT, SWT) were measured at both end diastole (d) and end systole (s). In DD group, LVID measured at the end of systole were significantly longer than that of ND (21.8 ± 2.9 *vs* 17.5 ± 0.1, mm, p < 0.05). As for LV wall thickness parameters (AWTd, AWTs, PWTs), they all showed a tendency to be thinner in the DD group than those in the ND group but still without statistical significance.

### Myocardial microstructure and ultrastructure findings

**Fig. 6:**
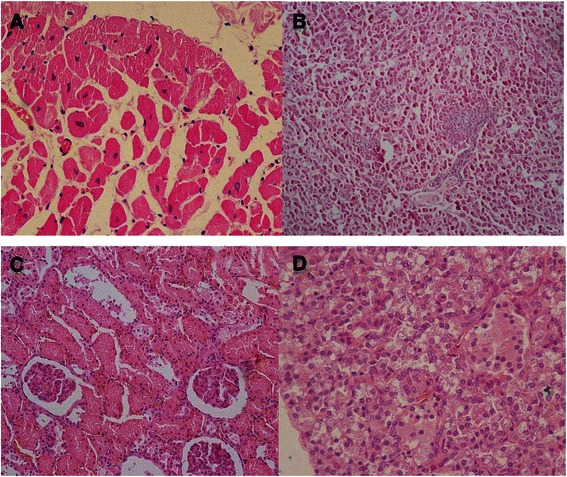
Myocardial microstructure and ultrastructure pictures observed by light scope and transmission electron microscope. **a**, **b**, **c** and **d** Myocardium, liver, kidney and pancreas pathological image of a DD monkey, respectively

**Fig. 7:**
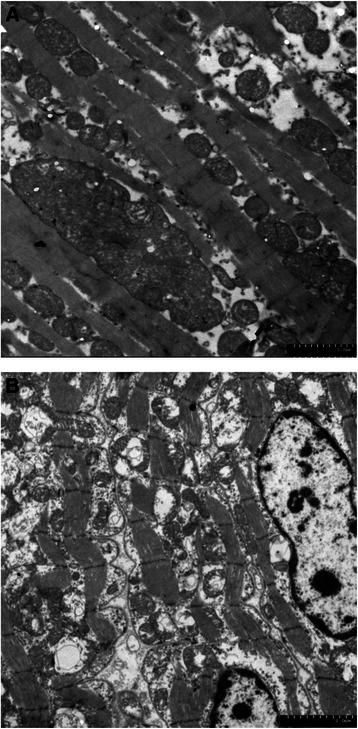
Myocardium ultrastructure TEM image of an ND monkey and a DD monkey. **a** ND monkey; **b** DD monkey

Pathologic analyses were used to assess the myocardial microstructure of a monkey with moderate LV diastolic dysfunction. H&E staining revealed myocardium swelling with granular degeneration and myocardial atrophy accompanying interstitial expansion (Fig. 6a). At the same time, lesions were found in other major organs: liver, kidney and pancreas (Fig. 6b, c and d). Firstly, cellular atrophy, hepatic sinusoid expansion, multifocal proliferation of the connective tissue and hepatic cords vanishment were found in the section of liver tissue. Secondly, shrinkage of the glomerulus, kidney tubules granular degeneration and sectional tubular epithelial detachment were observed in the section of kidney tissue. Thirdly, cellular swelling, decreasing cell numbers and cell shrinkage were observed in the section of pancreas tissue. Transmission electron microscopy was used to observe the mitochondrial ultrastructure of the hearts of a ND monkey and a monkey with moderate diastolic dysfunction (Fig. 7a and b). In the myocardial mitochondria of a ND monkey, mitochondria were regularly shaped and arranged between myofilaments with the cristae being clear and undamaged. However mitochondrial swelling and vacuolation, atrophy and rupture of myocardial fibers along with nuclear chromatin migration towards the cell’s edge are observed in the DD monkey images.

### Cardiac protein study

**Fig. 8:**
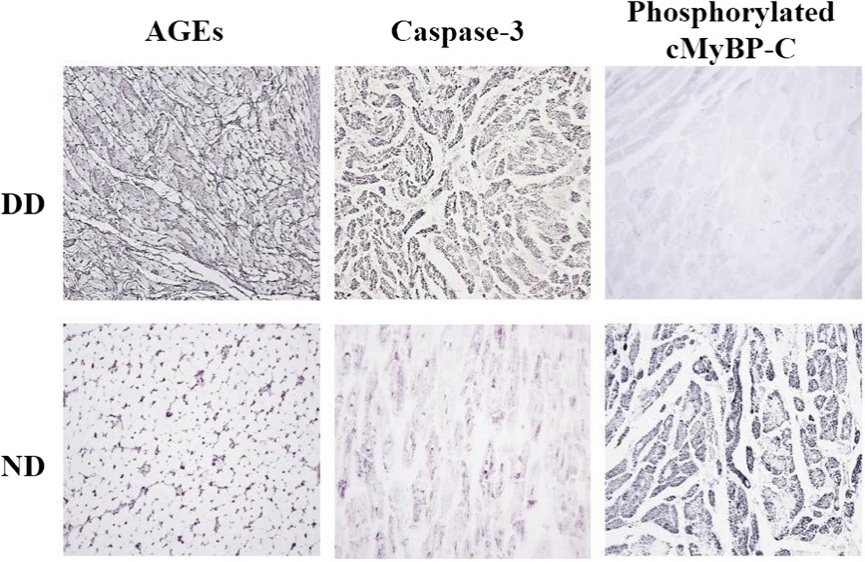
Immunohistochemistry stain results of AGEs, caspase-3 and cMyBP-C of an ND monkey and a DD monkey respectively

Immunohistochemical stains were used to assess the expression of different kinds of protein metabolized in the heart. AGEs, caspase-3 and phosphorylated cMyBP-C were stained by blue diaminobenzidine hydrochloride (DAB). Increased expression of AGEs, caspase-3 and decreased cMyBP-C phosphorylation in myocardium were observed by light-microscopic examination (Fig. 8), which were reflected by positive staining of these proteins throughout the field of microscope. However, there were no observable abnormal staining patterns as these in the sections of the ND monkey heart.

## Discussion

Echocardiography and CMR have been used for invasive cardiac phenotype assessment in diabetic animal models [15, 17, 18, 29] and humans [7, 8, 30–35]. Since there is no single diagnostic method for the identification of diastolic dysfunction, these tools can be complementary.

Echocardiography is an invasive and inexpensive tool that allows sonographers to evaluate changes in heart structure/function that are echogenic. This technique has been already used to assess the cardiac structure and function in non-human primates [36–39], all for establishing standardized echocardiographic reference values.

Mitral valve blood inflow measured by pulsed-wave Doppler and tissue Doppler imaging were combined to assess diastolic function in the hearts of monkeys. Normal pattern for diastolic function under pulsed-wave Doppler were defined as E/A > 1 in human. However, this value may also be observed at early stages of ischemia and hypertrophy. Doppler pattern of impaired LV relaxation, characterized by reduced early and increased late diastolic flow, is an early sign of DD (mild diastolic dysfunction) which shows E/A < 1 [40]. More advanced grades, manifested by predominant early diastolic filling and rapid velocity deceleration, known as restrictive filling patterns, cause an increase in filling pressure, lead to an increase in the E-wave, and result in a E/A ratio > 2 [41]. The hinge point of these grades is the intermediary values, the pseudonormal pattern or moderate DD group, has an E/A > 1 which results from an increase in the left atrial pressure [41] in the presence of a defective relaxation. The impaired LV relaxation leads to an increase in filling pressures in order to maintain normal cardiac output as a complement of the variables of mitral valve inflow velocity. A combination of mitral annulus velocity with peak e’ velocity < 8.5 cm/s and e’/a’ ratio < 1 was used to indicate relaxation abnormality, this mitral annulus velocity pattern could identify the underlying relaxation abnormality with a sensitivity of 88 % and specificity of 67 % [31]. Moreover, mitral annulus motion is less load dependent than conventional mitral inflow variables. This technique appears to be useful for evaluating diastolic function, especially in the detecting of a pseudonormal pattern. In this study, DD diagnosing and grading were referred to as a combination of parameters measured from pulsed-wave Doppler and TDI. Monkeys in ND group and T2DM with preserved diastolic function group showed that E/A > 1, e’ velocity < 8 cm/s, e’/a’ < 1 and E/e’ > 8. Subjects with E/A < 0.9 (impaired relaxation) and E/A > 1.3 (pseudonormal) were considered as mild DD and moderate DD, which were supported by TDI results such as decreased e’ velocity and e’/a’ < 1. The E/A ratio measured from T2DM monkeys in our trials was found to show a U-shaped prognostic behavior, which were similar to T2DM patients [7, 42].

Impairment of LV relaxation results in prolongation of the IVRT and EDT. In contrast, increasing filling pressure shortens IVRT and reduces EDT [43]. In this study, the mild DD group had the longest average EDT. Significant difference was found between the mild DD group and the T2DM with preserved diastolic function group. Though the difference of EDT between mild DD and the moderate DD groups were not significant, there was a tendency to register a decrease in the moderate DD group. As for the IVRT, no significances were found among groups. Heart rates (HR) of monkeys are usually twice or three times faster than that of the human being. During our echo study, heart rates ranged from 110 to 175 bpm when monkeys were sedated by ketamine. The wide variability of HR can influence the IVRT and EDT in evaluating the LV diastolic function.

Pulmonary venous flow pattern has also been used to distinguish normal from pseudonormal transmitral Doppler filling. Impairment of LV relaxation results in increased systolic (S) pulmonary venous flow, in contrast increasing filling pressure reduces PV systolic flow [43]. It has been reported that PVF indexes also change in a typical U-shaped pattern during the progression from normal to severe DD. In this study, the highest peak S and S/D ratio were seen in the mild DD group, whereas S was reduced in the moderate DD group and resulted in the reversal of S/D ratio (S/D < 1). As for AR velocity, differences between groups were unremarkable, which may be caused by the relative difficulty in obtaining accurate tracings by the transthoracic approach. Assisted by the TDI indexes, the PVF patterns seen provided more evidence of DD in these monkeys.

Decreased EF and FS were only seen in moderate DD subjects. It is still under debate as to which left ventricular function is impaired earlier in the evolution of diabetic cardiomyopathy. More studies stated that diastolic function is more commonly impaired than systolic and that diastolic dysfunction might be the initial abnormality in patients with the onset of diabetes [5, 7, 9, 42, 44, 45]. However, other researchers have recently shown that systolic strain alterations may exist despite normal diastolic function, which indicates systolic strain abnormality might be proposed as the first indicator of diabetic cardiomyopathy [46]. According to the results of the groupings in this study, significances of EF and FS were only found between the moderate DD group and ND group, indicating that diastolic dysfunction may be the earliest functional alteration in the course of diabetic cardiomyopathy, which lead to contractile abnormality with the progression of DD in spontaneous T2DM rhesus monkeys.

MRI provides accurate, reproducible, noninvasive representations of cardiac structure and function and measurements of regional myocardial wall thickening [47]. This non-invasive imaging tool has been proved to be powerful in evaluating the cardiac morphology of the diabetic rodent models [15, 17] and patients [35]. However, in vivo cine MRI studies on T2DM monkey models are very limited. In the present study, it was observed that T2DM rhesus monkeys with diastolic dysfunction showed significantly dilated LV internal diameter at end systole compared with ND monkeys. Though statistical differences were not seen, average LV internal diameter at end diastole were 12 % higher in DD subjects when compared with the ND group. These cardiac structural changes reflex the adaptive mechanism of reduced relaxation and contractility. As for left ventricular wall and septum thickness, no significant changes were seen between DD subjects and ND monkeys. Similarly, LV dilatation were accompanied by no significant changes in LV wall thickness as observed in diabetes rodents [48–50]. In this study, alterations of systolic function indicators were observed with the progression of DD by echocardiography, which included reductions in EF and FS. Increases in LV inner dimensions were also indicators of reduced contractile ability. Though significant changes were not found in LV wall and septal thickness, the average PWTd, PWTs and SWTs of the DD monkeys were 18.3 %, 11.9 % and 17.4 % all lower values than found in the ND ones. In a study using STZ treated rats [48, 50], it reported there is a gradual decrease in PWT after 8 weeks of diabetes while continuing after 12 weeks of recorded measurements. A relatively small sample number in this study may be a limitation in observing the differences between groups. Moreover, additional longitudinal studies are needed to trace the alteration with the progression of diabetes.

Histological abnormalities of myocardium were reported both in diabetic rodent models and human patients [51–53]. Characteristics of the pathological changes of the myocardium in the T2DM accompanying DD monkeys were consistent with the previous DCM studies. Meanwhile, lesions found in the liver, kidney and pancreas indicates that other diabetes complications also exist in these monkeys. Mitochondrial ultrastructure damage were also observed by TEM. Many laboratories have generated data suggesting that diabetic damage is a consequence of elevated production of reactive oxygen species (ROS) found at the mitochondrial respiratory chain during hyperglycemia [54, 55]. More and more laboratory findings support the hypothesis that mitochondrial ROS mediated the diabetes induced cardiac defects. Manifestations of mitochondria in our DCM rhesus monkeys were similar to those in previous rodent studies.

Assisted by immunohistochemistry stain, positive products were observed to be abnormally expressed in the DCM monkey myocardium compared with the ND monkey. The formation of AGEs on extracellular matrix components leads to accelerated increases in collagen cross linking that contributes to the myocardial stiffness in diabetes [10]. Apoptotic cell death occurs in the diabetic myocardium through the mitochondrial cytochrome c-mediated caspase-3 activation pathway, which plays a critical role in cardiac pathogenesis related to hyperglycemia [11]. Basal levels of cMyBP-C phosphorylation may be necessary for maintaining thick-filament orientation, dynamic regulation and contractile mechanics [12]. However, cMyBP-C phosphorylation was significantly decreased during the development of heart failure or pathologic hypertrophy. Phosphorylated cMyBP-C were lightly stained in the myocardium of the DCM monkey, which is indicative of contractile abnormality. Thus, myocardial impairments were observed at molecular level in abnormal organ functioning.

The spontaneously diabetic monkeys are of great importance in both biomedical investigation and for pharmaceutical studies. Literatures and our previous work demonstrated that the clinical manifestations of T2DM monkeys were highly similar to those of human beings. Results show that some if not most of our middle-aged rhesus monkeys were confirmed to have obesity, β-cell impairment, insulin level decline and persistent hyperglycemia verifying the development of spontaneous T2DM. The FPG of healthy monkeys in our monkey model is only approximately 10 %-15 % lower than that of humans. As for DD in monkeys diagnosed by echocardiography, relative parameters exhibit humanlike echo patterns with diastolic dysfunction progression, which has not been reported in rodent models. As for other features like MRI parameters, cardiac structure and biomarkers in cardiomyopathy, the monkeys share the same features as reported in human and rodents. Moreover, the development of spontaneous diabetes and its complications is a multifactorial slow progression. Of note, monkeys have a long enough life span that allows both disease progression and thorough longitudinal studies, which may shed light on the origins, pathways and pathogenesis of these pathologic changes. Meanwhile, these monkeys live in a highly controlled and stable environment with their metabolic history well documented. Thus long-term relationships between cardiovascular pathology changes and diabetic status can now be studied without the confounding lifestyle factors encountered in humans.

### Limitations of the study

The present study primarily demonstrated diastolic dysfunction and related myocardial impairments in spontaneous T2DM rhesus monkeys. Nevertheless, there are several limitations need to be addressed. Firstly, the echocardiographic parameters we examined in this study were not so sufficient as to allow us to draw a full picture of the dimension and function of the ventricle. It has been reported that EDV, ESV, and V_p_ obtained by color M-mode Doppler also change with cardiac dysfunction in a diabetic heart. Secondly, CMR can provide more information about cardiomyopathy by using late gadolinium enhancement (LGE), MR tagging and T1 mapping, which we unfortunately failed to obtain in the present study. We are planning to further investigate DCM in rhesus monkeys by CMR in the near future. In consideration of the maintenance of the model, we only sacrificed 2 monkeys for invasive measurements. Luckily, certain myocardial impairments were observed on both microstructure and ultrastructure levels. However, quantified measurements of these biomarkers need to be done on a larger scale for these animals to determine reliability. We are also preparing non-invasive assessments for a better understanding of the mechanisms of these biomarkers and more, hoping to provide evidence for the hypotheses proposed regarding the pathogenesis of DCM.

## Conclusions

The present study primarily demonstrated cardiomyopathy in spontaneous T2DM rhesus monkeys. Left ventricular diastolic dysfunction was diagnosed in six out of 15 T2DM monkeys, accompanied with structural abnormalities such as significantly increased LVID at end systole. Systolic related parameters, EF and FS, decreased with DD progression, however significance was found only between the moderate DD group and the ND group. Myocardial microstructure and ultrastructure damages were observed in a DD monkey. Meanwhile, the expression of DCM related proteins altered in a DD monkey. Characteristics of cardiomyopathy in spontaneous T2DM monkeys with DD are similar to those in DCM human patients. We will not only conduct more studies in these subjects for further investigations, but we will also screen for more diseased animals in the future to create a more effective drug testing platform.

### Ethics statement

The protocols were approved by the Institutional Animal Care and Use Committee (IACUC) of Sichuan PriMed Bio-Tech Group Co., Ltd.

## Abbreviations

E: Peak velocity of mitral blood flow during the rapid ventricular filling (cm/s)
A: Peak velocity of mitral blood flow during the atrial contraction (cm/s)
e’: Peak velocity of mitral annulus during the rapid ventricular filling (cm/s)
a’: Peak velocity of mitral annulus during the atrial contraction (cm/s)
S: Pulmonary venous flow peak systolic velocity (cm/s)
D: Pulmonary venous flow diastolic velocity (cm/s)
AR: Pulmonary venous flow atrial reversal velocity (cm/s)
EDV: End diastolic left ventricular volume (ml)
ESV: End systolic left ventricular volume (ml)
SV: Stroke volume (ml)
FS: Fractional shortening of the left ventricular (%)
EF: Ejection fraction (%)
SWTd: Septum wall thickness at end diastole (mm)
SWTs: Septum wall thickness at end systole (mm)
AWTd: Anterior wall thickness at end diastole (mm)
AWTs: Anterior wall thickness at end systole (mm)
PWTd: Posterior wall thickness at end diastole (mm)
PWTs: Posterior wall thickness at end systole (mm)
LVIDd: Left ventricular internal dimension at end diastole (mm)
LVIDs: Left ventricular internal dimension at end systole (mm)
LAAD: Left atrial anterior-posterior diameter (mm)
LATD: Left atrial transverse diameter (mm)
LAVD: Left atrial vertical diameter (mm).
